# Intrathecal B Cells in MS Have Significantly Greater Lymphangiogenic Potential Compared to B Cells Derived From Non-MS Subjects

**DOI:** 10.3389/fneur.2018.00554

**Published:** 2018-07-20

**Authors:** Jason Stein, Quangang Xu, Kayla C. Jackson, Elena Romm, Simone C. Wuest, Peter Kosa, Tianxia Wu, Bibiana Bielekova

**Affiliations:** ^1^Neuroimmunological Diseases Section, National Institute of Neurological Disorders and Stroke, National Institutes of Health, Bethesda, MD, United States; ^2^Department of Neurology, Chinese PLA General Hospital, Beijing, China; ^3^Clinical Trials Unit, National Institute of Neurological Disorders and Stroke, National Institutes of Health, Bethesda, MD, United States

**Keywords:** lymphangiogenesis, B cell immunology, multiple sclerosis, intrathecal inflammation, tertiary lymphoid follicles

## Abstract

Although B cell depletion is an effective therapy of multiple sclerosis (MS), the pathogenic functions of B cells in MS remain incompletely understood. We asked whether cerebrospinal fluid (CSF) B cells in MS secrete different cytokines than control-subject B cells and whether cytokine secretion affects MS phenotype. We blindly studied CSF B cells after their immortalization by Epstein-Barr Virus (EBV) in prospectively-collected MS patients and control subjects with other inflammatory-(OIND) or non-inflammatory neurological diseases (NIND) and healthy volunteers (HV). The pilot cohort (*n* = 80) was analyzed using intracellular cytokine staining (*n* = 101 B cell lines [BCL] derived from 35 out of 80 subjects). We validated differences in cytokine production in newly-generated CSF BCL (*n* = 207 BCL derived from subsequent 112 prospectively-recruited subjects representing validation cohort), using ELISA enhanced by objective, flow-cytometry-based B cell counting. After unblinding the pilot cohort, the immortalization efficiency was almost 5 times higher in MS patients compared to controls (*p* < 0.001). MS subjects' BCLs produced significantly more vascular endothelial growth factor (VEGF) compared to control BCLs. Progressive MS patients BCLs produced significantly more tumor necrosis factor (TNF)-α and lymphotoxin (LT)-α than BCL from relapsing-remitting MS (RRMS) patients. In the validation cohort, we observed lower secretion of IL-1β in RRMS patients, compared to all other diagnostic categories. The validation cohort validated enhanced VEGF-C production by BCL from RRMS patients and higher TNF-α and LT-α secretion by BCL from progressive MS. No significant differences among diagnostic categories were observed in secretion of IL-6 or GM-CSF. However, B cell secretion of IL-1β, TNF-α, and GM-CSF correlated significantly with the rate of accumulation of disability measured by MS disease severity scale (MS-DSS). Finally, all three cytokines with increased secretion in different stages of MS (i.e., VEGF-C, TNF-α, and LT-α) enhance lymphangiogenesis, suggesting that intrathecal B cells directly facilitate the formation of tertiary lymphoid follicles, thus compartmentalizing inflammation to the central nervous system.

## Introduction

Multiple sclerosis (MS) is a chronic inflammatory demyelinating disease of the central nervous system (CNS) thought to be mediated by myelin-specific CD4+ T cells (1, 2). Whereas depletion of CD4+ T cells does not reduce formation of MS lesions (3), depletion of B cells is strikingly more effective (4). Moreover, B cell depletion inhibits disability progression in MS subjects younger than 55 years, including those with progressive MS (PMS) (5, 6).

Indeed, intrathecal activation of B cells and their differentiated, antibody (Ab)-producing counterparts (plasmablasts and plasma cells) is the hallmark of MS (7, 8). Pathologists identified B cells in the meningeal tissue, in the form of diffuse infiltrates in most MS subjects (9) or tertiary lymphoid follicles in some secondary-progressive MS (SPMS) patients (10, 11). The same authors also demonstrated that meningeal follicles harbor Epstein-Barr Virus (EBV) (12, 13), a finding not reproduced by others (14). Nevertheless, EBV remains one of the best documented environmental links in MS (15) with a possible causal role (16, 17).

Despite this wealth of observational data, it remains unclear whether B cell depleting therapies must also deplete intrathecal B cells, in addition to peripheral B cells, to achieve their efficacy (18, 19). Similarly, the exact functions by which intrathecal B cells modulate MS severity or phenotype are unknown.

Analysis of intrathecal B cell functions, whether it be their antigen-presenting ability, the ability to co-stimulate T cells, or produce soluble factors, has been precluded by low numbers of B cells in the cerebrospinal fluid (CSF) (20). With current technologies, it is possible to isolate only few hundred B cells from a single MS patient lumbar puncture. Thus, the phenotypic analyses of CSF B cells performed so far focused on a limited number of surface markers (7, 8, 21, 22). These studies demonstrated a significantly higher frequency of CSF B cells and plasmablasts in patients with MS and other inflammatory neurological diseases (OIND) in comparison to non-inflammatory neurological diseases (NIND) controls (7, 8).

To examine the functional properties of intrathecal B cells, we optimized a procedure for the transformation of CSF B cells by EBV (23) and blindly analyzed cytokine secretion from a large number of intrathecal B cell lines (BCL) derived from pilot and validation cohorts, described in this paper.

## Methods

### Patients

This study was carried out in accordance with the recommendations of the institutional review board (IRB) at the National Institutes of Health (NIH). The protocol was approved by the NIH Combined Neuroscience IRB, and all subjects gave written informed consent in accordance with the Declaration of Helsinki. We applied the optimized EBV-transformation procedure to prospectively-acquired untreated patients presenting for a diagnostic work-up of CNS neuroimmunological disorders or for screening to NIH PMS clinical trials under the NIH protocol “Comprehensive multimodal analysis of neuroimmunological diseases of the central nervous system” (ClinicalTrials.gov identifier NCT00794352; Table 1; Supplemental Information). Eligible subjects were age 18–75 with clinical and neuroimaging evidence of immune-mediated CNS damage or healthy volunteers (HV) with no CNS disease and no risk factors for immune-mediated systemic or CNS diseases. Patients underwent a diagnostic workup as previously detailed (24), and MS was diagnosed based on 2010 revisions to McDonald diagnostic criteria (25).

**Table 1:** Demographic, clinical, and CSF data of pilot and validation cohort patients.

	**HV**	**NIND^*a*^**	**OIND^*b*^**	**RRMS**	**SPMS**	**PPMS**	**All MS**
***N***^♢^
Pilot		19 (3)	14 (4)	35 (25)	3 (0)	9 (3)	47 (28)
Validation	4	4	25	26	24	29	79
**Age at baseline**, **year**
Pilot		47 (28–66)	41 (30–62)	40 (24–65)	56 (49–65)	51 (47–61)	47 (24–65)
Validation	46 (23–62)	45 (23–62)	50 (20–78)	38 (18–65)	50 (27–64)	53 (31–64)	47 (18–65)
**Gender, F/M**
Pilot		13/6	6/8	15/20	2/1	2/7	19/28
Validation	2/2	4/0	13/12	15/11	12/12	9/20	36/43
**EBVCA-IgG (% EBV seropositive/% Missing)**
Pilot		100/33	100/77	100/11	100/0	100/0	100/9
Validation	100/75	100/50	100/72	100/8	100/21	100/0	100/9
**% Treated at time of LP**
Pilot		0	0	24	33	0	20
Validation	0	0	0	10	4	14	11
**CSF WBC, cells/**μ**L**
Pilot		1 (0–7)	4 (1–19)^d^	4 (0–19)^d^	0 (0–1)	2 (0–8)	2.5 (0–19)^c^
Validation	2 (1–2)	1 (0–3)	27 (0–318)	6 (0–21)	4 (0–27)^e^	4 (0–13.5)	5 (0–27)^e^
**CSF Protein, mg/dL**
Pilot		34 (24–51)	46 (25–71)^c^	41 (26–97)	53 (40–61)^d^	44 (26–63)^c^	43 (26–97)^c^
Validation	42 (34–56)	43 (36–53)	88 (26–301)	42 (28–73)^e^	45 (26–81)	50 (24–127)	46 (24–127)
**CSF Glucose, mg/dL**
Pilot		55 (48–69)	63 (50–88)	59 (50–77)^c^	60 (55–63)	60 (54–67)^c^	59 (50–77)^c^
Validation	62 (60–66)	53 (47–58)	51 (21–94)	57 (53–59)	59 (51–70)	60 (49–74)^f^	58 (49–74)^f^
**CSF IgG Index**
Pilot		0.50 (0.38–0.59)	0.72 (0.48–2.49)^d^	0.90 (0.52–5.30)^d^	0.59 (0.50–0.68)^c^	0.79 (0.60–2.85)^d^	0.83 (0.50–5.30)^d^
Validation	0.5 (0.37–0.59)	0.48 (0.45–0.55)	0.60 (0–2.49)	0.96 (0.53–2.81)^f^	1.21 (0.48–2.36)^f^	1.07 (0.42–2.58)^f^	1.08 (0.42–2.58)^f^

♢ *In the pilot cohort, numbers in parentheses represent the number of patients with ≥1 immortalized BCL. In the validation cohort, numbers represent only patients with ≥1 successful BCL transformation*.

a *NIND, non-inflammatory neurological diseases, including seizures and ischemic cerebrovascular disease*.

b *OIND, other inflammatory neurological diseases, including CNS sarcoidosis, systemic lupus erythematosus with CNS involvement, neuromyelitis optica, acute disseminated encephalomyelitis, lymphocytic encephalitis, progressive encephalopathy and transverse myelitis*.

c *p < 0.05 compared to NIND group*.

d *p < 0.01 compared to NIND group*.

e *p < 0.05 compared to HV+NIND group*.

f *p < 0.01 compared to HV+NIND group*.

### Generation of EBV-transformed BCL

All prospectively-enrolled subjects were coded, and CSF samples were processed blindly. CSF cells were extracted by centrifugation (300 g × 10 min) within 30 min of CSF collection. EBV immortalization Protocol A was used in the pilot cohort between June 2008 and June 2010 on all subjects who lacked blood contamination (i.e., < 100 RBC/μL of CSF) and had a minimum of 5000 CSF leukocytes available for immortalization. Protocol B was used in the validation cohort collected between July 2010 and February 2014 on subjects without blood contamination who had a minimum of 10,000 CSF leukocytes for immortalization.

### Protocol A: protocol studying immortalization efficacy in the pilot cohort

Irradiated (6,000 Rd) CD40+ NIH 3T3 cells were seeded on a 384-well plate (5,000 cells per well) 1 day before scheduled CSF acquisition to achieve their confluency. CSF cells were counted, and co-cultured with CD40+ NIH 3T3 cells at 5,000 CSF cells/well in B cell media (RPMI1640 + 15% FBS + Penicillin/Streptomycin + L-glutamine) containing 2.5 μg/mL CpG oligonucleotides (ODN 2006, InvivoGen, San Diego, USA), 3 ng/mL IFN-γ, 0.5 μg/mL cyclosporine A (Sigma-Aldrich, St. Louis, USA), and 50% EBV supernatant extracted from a confluent B95.8 marmoset leukocyte cell line (CRL-1612, ATCC, Manassas, USA). These optimized conditions for CSF B cell immortalization were determined in extensive preliminary experiments using protocols previously described for immortalization of peripheral B cells (26).

For subjects with larger numbers of CSF cells, proportionally more wells were seeded at 5,000 cells/well.

Growing cell clusters were transferred after 2–3 weeks to progressively larger volumes of fresh B cell media until approximately 10 million immortalized CSF B cells were obtained. These were then cryopreserved. Functional analyses were performed in parallel for all immortalized BCL.

### Protocol B: modification to increase immortalization rate of non-MS subjects

Instead of seeding multiple wells at 5,000 CSF cells/well, for validation cohort we seeded only 1–3 wells/subject, with a 10,000–25,000 CSF cells/well of 384-well plate (i.e., increasing the number of CSF cells for subjects with a larger number of available CSF cells).

### Immunophenotyping by fluorescence-activated cell sorting (FACS; pilot cohort)

Thawed, overnight-rested BCL were evaluated for surface expression of costimulatory and activation markers. Intracellular cytokine staining (ICCS) was performed as described (27). Briefly, BCL were activated with phorbol 12-myristate 13-acetate (PMA; 32 nM) and Ionomycin (1 μM) in the presence of Brefeldin A (1 μL per 1 × 10^6^ cells, GolgiPlug, BD Biosciences, San Jose, USA) for 12–14 h. After staining for surface markers, BCL were fixed, permeabilized, and stained for intracellular cytokines. Data were acquired by LSR II and analyzed by FACSDiva software (both BD Biosciences).

The following mouse anti-human monoclonal antibodies were used for BCL phenotypization: APC-α-CD19 (HIB19, BD, Franklin Lakes, USA); PE-α-CD5 (UCHT2, BD); FITC-α-CD24 (ML5, BD); APC H7-α-CD25 (MA251, BD); Alexa Fluor 700-α-CD38 (HIT2, eBioscience, San Diego, USA); CD80 (L307.4, BD); PE-α-CD86 (IT2.2, eBioscience); PE-α-IL4 (8D4-8,BD); PE-α-IL6 (MQ2-6A3, BD); PE-α-IL12 p40/p70 (C11.5, BD); PE-α-LT α (3598111, BD); APC-α-VEGF (23410, R&D Systems Inc., Minneapolis, USA); APC-α-TGF-β1 (27232, R&D Systems Inc.); APC-α-IFN-γ (B27, BD); APC-α-TNF-α (MAb11, BD). We also used rat anti-human monoclonal antibodies: PE-α-IL2 (MQ1-17H12, BD) and eFluor 450-α-IL10 (JES3-9D7, eBioscience).

### B cell activation for enzyme-linked immunosorbent assay (ELISA) assays (validation cohort)

Cryopreserved CSF BCL were thawed and cultured for 1 week in B cell media: RPMI-1640, 1% penicillin/streptomycin, 1% gentamicin, 1% L-glutamine (all Lonza, Basel, Switzerland), and 15% fetal bovine serum (Gemini Bio-products, West Sacramento, USA). Cells were then resuspended in X-VIVO media (Lonza), counted by hemocytometer (LW Scientific, Lawrenceville, USA), and resuspended at 1 × 10^6^ cells/mL. This aliquot was also used for proportional live-B cell counting using flow cytometry (see below). Remaining cells were seeded in 24 well-plate at 1 × 10^6^ B cells/well either unstimulated (control) or stimulated with PMA (5 nM) and ionomycin (0.5 μM; concentrations optimized in preliminary experiments to maximize cytokine production). After 18 h of culture, the supernatants were centrifuged, aliquoted and frozen at −80°C until ELISA assay.

### Proportional flow cytometry-based counting of B cells for ELISA assays (validation cohort)

**Figure 1:**
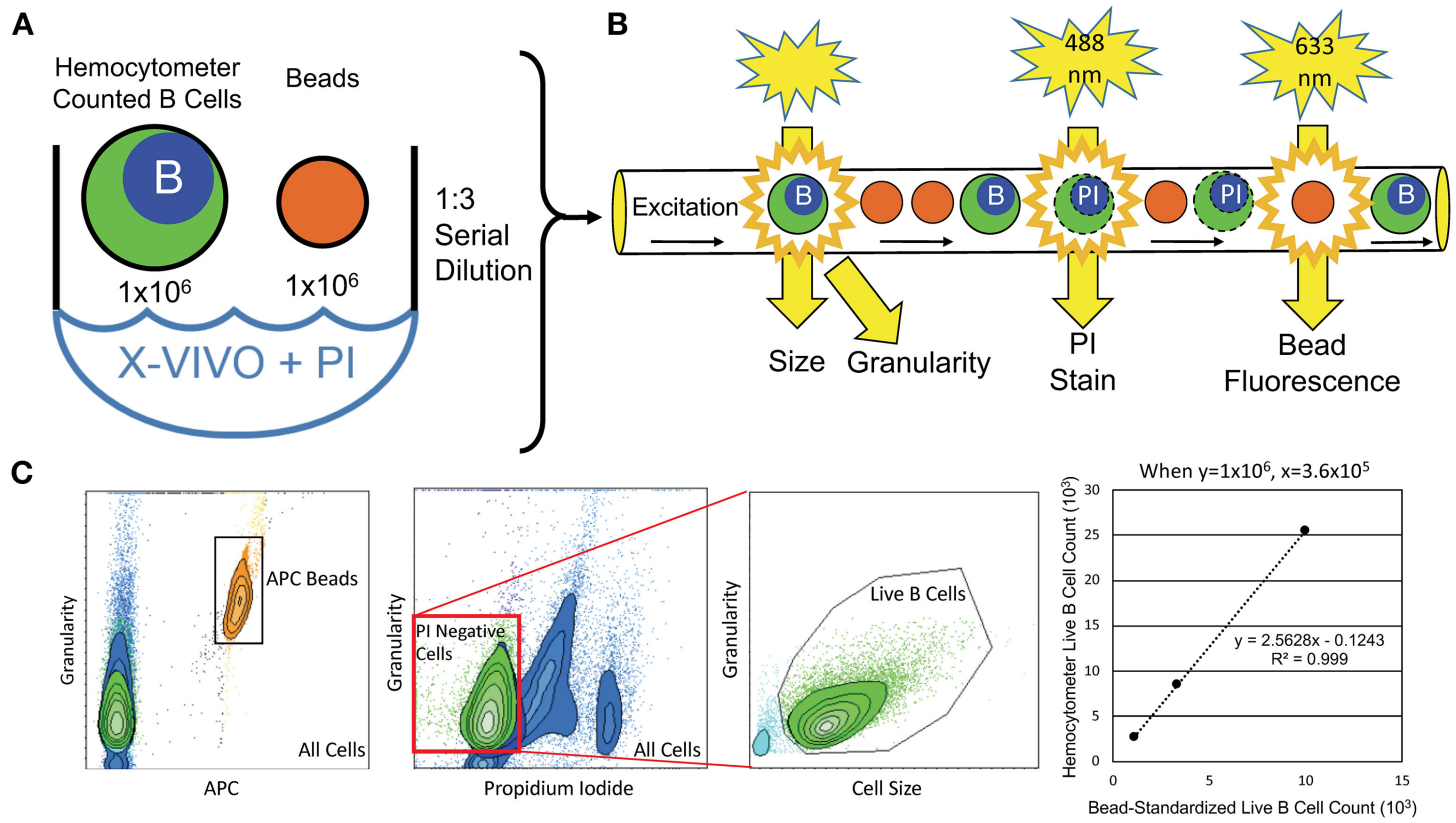
Flow-cytometry-based procedure for objective enumeration of live CSF B cells that contributed to supernatants analyzed for cytokine concentrations by ELISA in the independent validation cohort. **(A)** Coded, *in-vitro* expanded EBV-immortalized CSF BCL were manually counted and resuspended at 1 × 10^6^ B cells/mL. This B cell dilution was then used to seed plates for cytokine detection, while, simultaneously, 1 × 10^6^ B cells from this aliquot were stained with PI and mixed with 1 × 10^6^ fluorescently (APC)-tagged microbeads (same batch used for the entire validation cohort) for flow cytometry analysis. **(B)** B cell/microbead mixture was serially diluted three times at 1:3, before their proportional enumeration by flow cytometry. **(C)** Flow cytometry output quantified microbeads based on APC fluorescence signal and B cells based on size and granularity. Live B cells in cultures were gated as PI-negative, as dying B cells intercalate PI stain into DNA, altering their emission profile. Numbers of live B cells were plotted against APC microbeads for all 3 dilutions to derive patient-specific linear regressions, from which exact number of live B cells seeded and activated in cytokine-secretion assays was calculated, based on known (and equal among all subjects in the validation cohort) number of fluorescent microbeads.

To objectively quantify the number of live B cells that generated supernatants for ELISA, we developed flow-cytometry-based assays (Figure 1). An aliquot of hemocytometer-counted BCL at 1 × 10^6^ cells/mL was resuspended in propidium iodide (PI; 2 μg/mL) to stain dying/dead cells. One million APC Calibration fluorescent particles (SPHEROTM 5.0–5.9 μM; Spherotech, Lake Forest, USA) were added to each BCL to proportionally enumerate BCL between different subjects. One lot of beads was used for the entire study. Serial dilutions of the PI-stained BCL with APC beads mixture were analyzed by flow cytometer to derive linear regression models of live (PI negative) B cells per APC beads, which were utilized for proportional normalization of B cells between different subjects/experiments (Figure 1).

### ELISA assays

Supernatants from stimulated CSF BCL were analyzed for interleukin (IL)-1β, IL-6, IL-10, tumor necrosis factor (TNF)-α, lymphotoxin (LT)-α and granulocyte macrophage colony stimulating factor (GM-CSF) using the V-Plex Meso Scale Discovery platform ELISA (Meso Scale Diagnostics, Rockville, USA) per manufacturer protocols (28). Additionally, we developed vascular endothelial growth factor (VEGF)-A and VEGF-C assays *de novo* using antibodies from R&D Systems Inc. Table 2 defines each assay, manufacturer, detection limits, and intra-assay variability. Pilot studies of each assay suggested that supernatants from unstimulated (control) B cells do not contain measurable levels of cytokines. Therefore, stimulated BCL conditions were analyzed exclusively.

**Table 2:** ELISA assay development criteria.

**Molecule**	**Manufacturer (antibody clone, kit)**	**CSF dilution factor**	**Detection limit (pg/mL)^a^**	**Inter-assay coefficient of variance (%)^b^**
IL-1β	Meso scale diagnostics (K15050D)	2	0.17	9.35
IL-6	Meso scale diagnostics (K15050D)	2	2.11	17.94
TNF-α	Meso scale diagnostics (K15050D)	10	2.12	39.04
LT-α	Meso scale diagnostics (K15050D)	20	1.16	9.46
GM-CSF	Meso scale diagnostics (K15050D)	8	1.08	15.02
IL-10	Meso scale diagnostics (K151TZK)	4	0.45	5.80
VEGF-A	R&D systems (MAB293, DY293B)	1	1.08	12.10
VEGF-C	R&D systems (MAB752, DY752B)	1	522.97	29.40

a *Detection limit determined from linear portion of standard curve. Limit is recalculated to reflect utilized dilution factor of CSF*.

b *Intra-assay coefficient of variance was not calculated because samples were not run in duplicate*.

### Statistical analysis

The diagnostic code (i.e., relapsing-remitting MS [RRMS], SPMS and primary-progressive MS [PPMS], HV, NIND, and OIND) was broken only after the functional data were acquired.

Gender spread between diagnostic categories was evaluated with a chi-square test. Differences in demographic, clinical and CSF data in each diagnostic category were assessed using a Kruskal-Wallis analysis of variance on ranks, followed by Dunn's test with Dunn-Šidák adjustments for multiple comparisons, while differences in EBV transformation rate were assessed with the binomial proportion test. Between-group comparisons in the pilot cohort were assessed using a Kruskal-Wallis analysis of variance on ranks followed by the multiplicity adjusted Dunn's multiple comparisons test. For the validation cohort, we fit a linear mixed model with diagnosis as a fixed effect and subject as a random effect. This allowed us to test for differences between diagnostic groups while accounting for BCLs that were generated from the same CSF sample. We assessed group differences in the mixed model using an analysis of variance (ANOVA) followed by pair-wise comparisons of the least-squares means for the model with a Tukey adjustment for pair-wise multiple comparisons. When no differences between diagnostic categories were observed (verified with one-way *t*-tests to assess group-wise differences that resulted in *p* > 0.05), some were merged logically: for the pilot cohort, we merged OIND and NIND into other neurological diseases (OND). In the validation cohort, we merged HV+NIND, and we combined PPMS and SPMS to PMS cohorts. Box-Cox transformation was applied to the biomarker variables with a non-normal distribution. The Shapiro-Wilk test was used to test the normality of the residuals. SAS version 9.4, Graphpad Prism version 7.0b, and R version 3.4.3 were used for the above analyses and *p* < 0.05 was used as the significance level.

Correlations between cytokines in the pilot cohort were assessed by Pearson correlation coefficients. In the validation cohort, we used Spearman correlation coefficients with a Bonferroni *p*-value adjustment to assess the correlation between cytokine concentrations and the MS disease severity scale (MS-DSS) (29), a new sensitive measure of MS disease severity.

## Results

After unblinding the diagnostic codes, the *n* = 80 pilot cohort consisted of 47 MS patients (35 RRMS, 9 PPMS and 3 SPMS) and 33 controls (14 OIND and 19 NIND). MS and controls were well matched for demographic data (Table 1). All subjects with available EBV serology were found to be seropositive (Table 1).

### EBV transformation rate

The average number of CSF cells seeded per patient was similar between MS and controls (39,623 ± 39,207 vs. 39,412 ± 76,718; ns). Likewise, the number of seeded wells per patient was comparable between the two cohorts (3.87 ± 3.02 vs. 2.55 ± 2.63; ns). We generated at least 1 BCL in 28 out of 47 MS patients (59.6%). In contrast, we obtained CSF BCL in only 7 out of 33 OND controls (21.2%; *p* = 0.004 in comparison to MS). We quantified transformation efficiency as the number of seeded CSF cells required to obtain one BCL. The transformation efficiency in the control group was, on average, 1 BCL per 100,046 seeded CSF cells (1/91,130 in OIND and 1/129,767 in NIND; ns). Transformation efficiency was almost 5 times higher in the MS group, reaching 1 BCL per 21,163 seeded CSF cells (*p* < 0.001).

The efficiency of immortalization correlated weakly with CSF cell count (Spearman ρ = 0.337, *p* = 0.00299) and with CSF IgG index (Spearman ρ = 0.391, *p* = 5 × 10^−4^).

### General phenotype of CSF BCL

**Figure 2:**
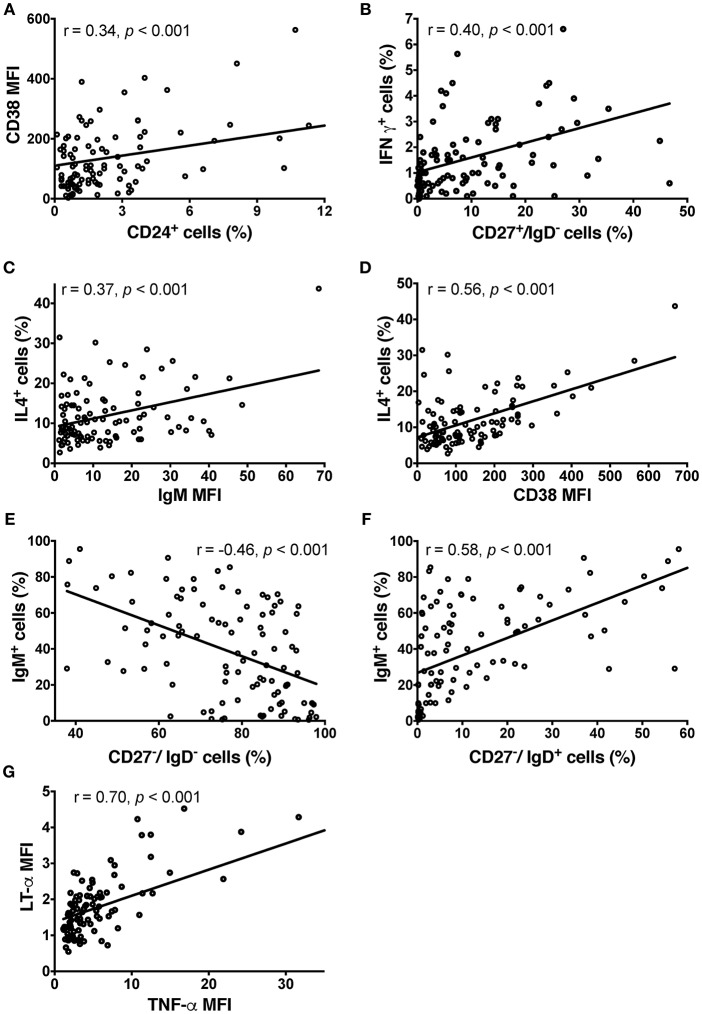
Phenotype of CSF BCL in the pilot cohort detected by ICCS. **(A)** Relationship between surface expression of CD38 and CD24+ cells. **(B)** IFN-γ production resides mostly in switched memory B cells based on significant correlation between proportion of CD27+/IgD– cells and BCL production of IFN-γ. In contrast, transitional B cells are the main producers of IL-4 based on a significant correlation between the proportion of IL-4-producing B cells and IgM **(C)** and CD38 **(D)** in BCL. IgM and CD38 are co-expressed in transitional B cells. **(E)** The negative correlation between proportion of IgM-expressing B cells and double negative (CD27–/IgD–) B cells in CSF BCL support the conclusion that double negative B cells are tissue-resident memory B cells, rather than naïve, IgM expressing B cells. **(F)** Instead, the proportion of naïve B cells (CD27–/IgD+) positively correlates with IgM expression. **(G)** LT-α and TNF-α are strongly co-expressed, based on their strong, positive correlation in individual BCL. Group differences were assessed using Kruskal-Wallis analysis of variance on ranks followed by an adjusted Dunn's test for multiple comparisons (*R*), Pearson correlation coefficients; MFI, Mean Fluorescence Intensity.

CSF BCL did not express high levels of CD5 (1.67 ± 7.94%), CD24 (2.7 ± 3.65%) or CD25 (7.91 ± 7.9%). BCL were almost 100% positive for CD38 (90.52 ± 15.45%) and all expressed co-stimulatory molecules CD80 and CD86. We observed a significant correlation between the surface expression of CD24 and CD38 (Pearson *r* = 0.344, *p* = 4.26 × 10^−4^; Figure 2A), which reflects their synchronous expression in transitional B cells (30).

Out of the 10 cytokines examined, none of the BCL produced detectable levels of IL-2, IL-6, IL-10, IL-12 or transforming growth factor (TGF)-β and only few BCL produced low levels of interferon (IFN)-γ. IFN-γ production resided mostly in class-switched memory B cells (CD27+/IgD–), as we observed significant correlation between proportion of CD27+/IgD– cells in EBV-transformed BCL and proportion of IFN-γ+ cells (Pearson *r* = 0.403, *p* = 2.96 × 10^−4^; Figure 2B).

The majority of BCL produced detectable levels of IL-4, TNF-α, LT-α, and VEGF. IL-4 secretion resided mostly in naïve and transitional B cells, as proportion of IL-4+ B cells in EBV-transformed BCL correlated moderately with IgM expression (Pearson *r* = 0.37, *p* = 1.4 × 10^−4^; Figure 2C) and highly with CD38 expression (Pearson *r* = 0.56, *p* = 1.41 × 10^−9^; Figure 2D). These naïve and transitional B cells represented only a minority (12.8%) of CSF BCL. The remaining cytokines were not preferentially expressed by a distinct B cell phenotype. The majority of CSF B cells (87.2%) were memory B cells, including CD27-/IgD- memory tissue-based B cells (31), expressing CD80 and CD86 cell activation markers, but generally lacking IgM expression (Pearson *r* = −0.46, *p* = 1.38 × 10^−6^; Figure 2E), which is instead a hallmark of CD27–/IgD+ naive B cells (Pearson *r* = 0.58, *p* = 3 × 10^−10^; Figure 2F). TNF-α and LT-α were strongly co-expressed by the same B cells, as evidenced by strong correlations between them (Pearson *r* = 0.70, *p* = 6.033 × 10^−16^; Figure 2G).

### Inter-group differences in the phenotype of CSF BCL

After unblinding the diagnostic codes, we observed that only 13 BCL were derived from control patients (3 from NIND, 10 from OIND), 15 BCL were from patients with PPMS and the remaining 73 BCL were from patients with RRMS.

**Figure 3:**
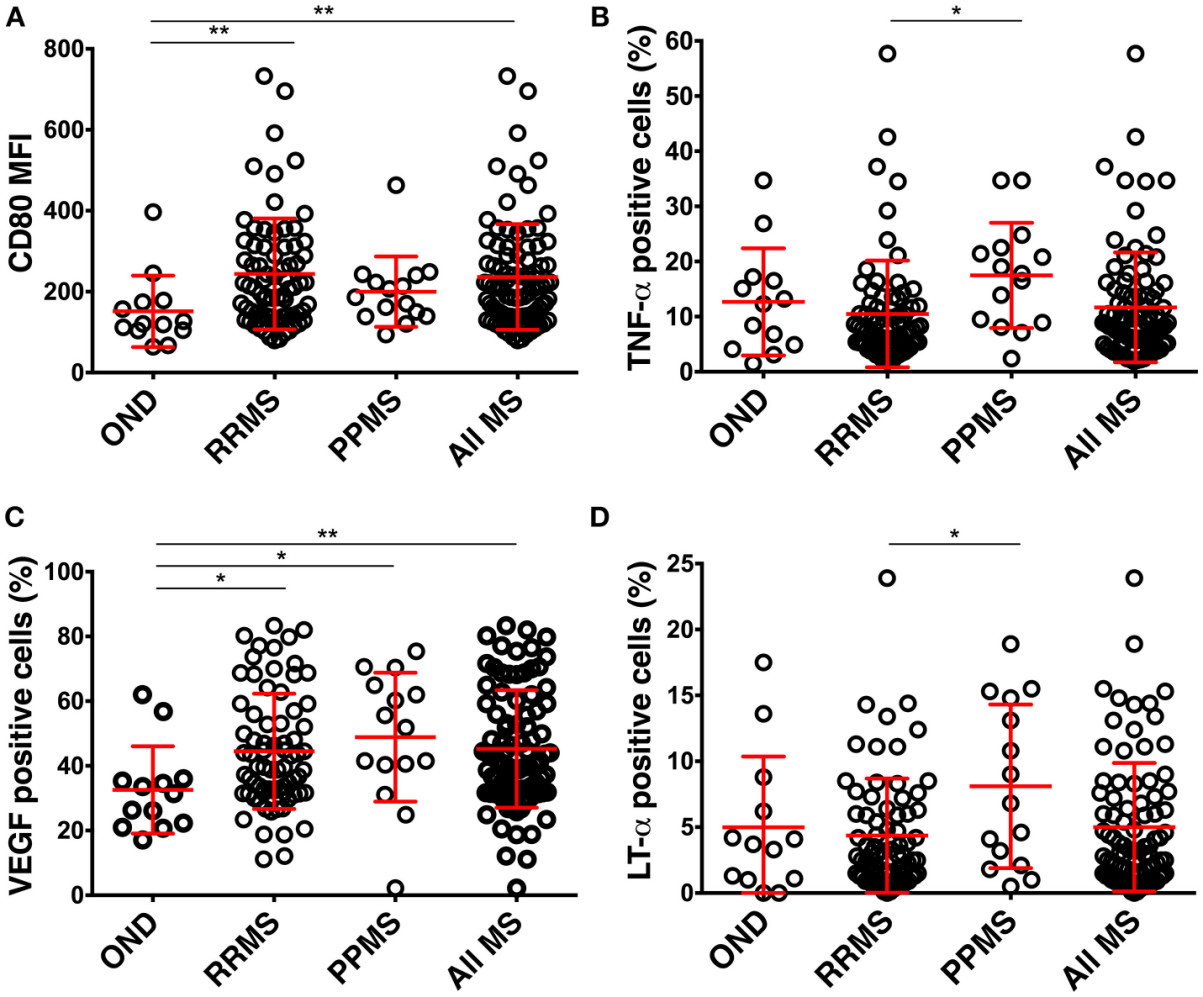
Differences in the phenotype of CSF BCL in the pilot cohort among diagnostic categories. **(A)** BCL from RRMS (*n* = 73) and all MS patients express higher CD80 than BCL from controls (OIND *n* = 10; NIND *n* = 3). **(B)** BCL from MS (*n* = 88) patients produce more VEGF than BCL from controls. **(C,D)** BCL from PPMS (*n* = 15) patients produce more TNF-α and LT-α than BCL from RRMS. Red lines represent means and positive and negative standard deviation from the mean for each diagnostic group. *p < 0.05, **p < 0.01; MFI, mean fluorescence intensity).

BCL from all MS patients had a significantly higher expression of CD80 in comparison to BCL from controls (Figure 3A). No other differences in surface markers reached statistical significance.

Similarly, the only significant difference between MS patients and controls resided in the higher production of VEGF in BCL derived from MS patients (Figure 3B). Finally, BCL derived from PPMS produced significantly higher amounts of TNF-α and LT-α as compared to BCL from RRMS patients (Figures 3C,D).

### Validation cohort

The limitation of the pilot cohort was significantly lower EBV transformation efficiency of the control samples, which we did not anticipate. This led to small numbers of control BCL. Similarly, the numbers of BCL derived from PMS subjects were low, which raised a possibility that the statistically significant increases in the secretion of TNF-α and LT-α we have observed in this cohort may have been over-estimated (32). Therefore, we modified EBV immortalization method to enhance its efficacy by seeding 10,000–25,000 CSF cells into a single well (Protocol B, see section Methods) for the independent validation cohort. This precluded us from validating the differences in EBV immortalization efficiency, but this observation from the pilot cohort had a very low *p*-value and therefore low Type II error. Instead, the goal of the validation cohort was to transform sufficient number of BCL from MS controls and PMS patients to disprove or validate differences in VEGF, TNF-α, and LT-α secretion among MS and controls and the relationship of these cytokines to the MS phenotype.

We also decided to use ELISA instead of ICCS we employed in the pilot cohort because a single ICCS protocol cannot be optimized for all cytokines simultaneously, as some cytokines are secreted early after activation while others are secreted late and they have different sensitivity to protein transport inhibitors such as monensin and brefeldin A. We hypothesized that our inability to detect some cytokines commonly secreted by B cells (such as IL-10 or IL-6) in the pilot cohort was a consequence of this technical problem of ICCS. ELISA eliminates this problem, but it poses another: the results are highly dependent on the exact number of seeded cells. Thus, instead of relying solely on manual cell counts, we implemented an unbiased flow-cytometry-based normalization of detected cytokine concentrations to live B cell numbers (Figure 1 and section Methods) among all tested subjects. In addition to studying VEGF (A and C isoforms were studied separately) and TNF-α, LT-α, IL-6, and IL-10 to refute or validate findings from the pilot cohort, we also analyzed *de novo* two additional cytokines with putative role in MS, IL-1β, and GM-CSF (33).

### Cytokine secretion of CSF B cells in the independent validation cohort

**Figure 4:**
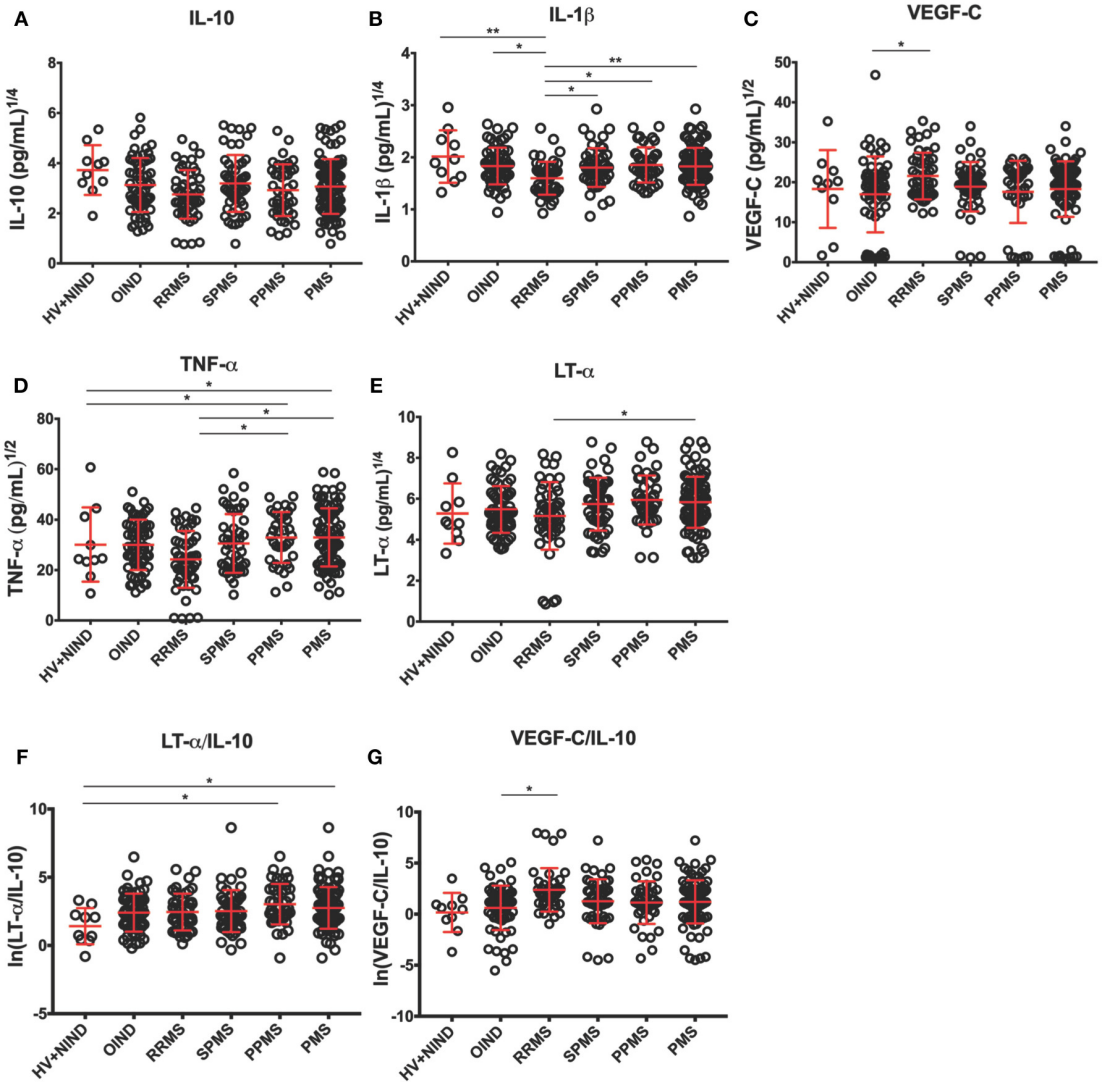
Differences in CSF BCL cytokine secretion among diagnostic categories in the independent validation cohort. **(A–E)** Depict concentrations of cytokines normalized to 1 million of live seeded B cells, while **(F,G)** depict ratios of pro-inflammatory cytokines to immunoregulatory IL-10 that reached statistical significance after adjustment for multiple comparisons. Superscripts in the y-axis correspond to lambda values of Box-Cox statistical normalization test **(A–E)**, while ln signifies natural logarithm of cytokine concentration values **(F,G)**. Dots represent individual BCL concentration (or ratio of cytokine concentration). Red lines indicate mean with positive and negative standard deviation for each diagnostic group. Group differences were assessed using an ANOVA on a linear mixed model with subject specified as a random effect. ^*^p < 0.05, ^**^p < 0.01 (after adjustments for multiple comparisons).

Table 3 describes the observed cytokine secretion results after unblinding diagnostic groups in the validation cohort (Figure 4G).

**Table 3:** Phenotype of validation group CSF BCL: secreted cytokine expression.

**Cytokine**	**Diagnostic category**	**Concentrations (pg/mL) mean SD median**			***F*-test *p***	**Pair-wise comparison**	**Tukey adjusted *p***	**Difference between means (pg/mL)**
IL-1β	HV+NIND	22.6	22.6	13.9	0.0011	HV+NIND vs. RRMS	0.0031	14.4
	OIND	13.9	10.8	10.3		OIND vs. RRMS	0.0434	5.7
	RRMS	8.2	7.3	7.2		RRMS vs. SPMS	0.0377	5.3
	SPMS	13.5	12.6	9.6		RRMS vs. PPMS	0.0111	6.1
	PPMS	14.3	10.7	11.2		RRMS vs. PMS	0.0028	5.6
	PMS	13.8	11.73	10.1				
IL-6	HV+NIND	232.4	268.1	91.5	0.3774			
	OIND	172.6	278.5	62.2				
	RRMS	107.8	141.1	72.8				
	SPMS	143.1	240.3	55.0				
	PPMS	195.6	294.0	82.1				
	PMS	167.1	266.0	69.9				
TNF-α	HV+NIND	1099.9	1081.7	601.6	0.0044	HV+NIND vs. PPMS	0.0242	−184.0
	OIND	1000.4	604.9	865.5		HV+NIND vs. PMS	0.0217	−117.0
	RRMS	713.8	538.9	582.1		RRMS vs. PPMS	0.0193	−570.1
	SPMS	1160.5	876.1	904.8		RRMS vs. PMS	0.0122	−503.1
	PPMS	1283.9	686.7	1254.9				
	PMS	1216.9	793.4	1039				
GM-CSF	HV+NIND	194.1	230.8	99.4	0.1827			
	OIND	203.3	339.9	75.6				
	RRMS	240.5	526.3	52.4				
	SPMS	428.6	813.9	100.7				
	PPMS	356.6	534.2	126.0				
	PMS	395.6	697.4	121.4				
LT-α	HV+NIND	1158.7	1399.7	548.4	0.0314	RRMS vs. PMS	0.023	−389.0
	OIND	1149.6	976.5	784.3				
	RRMS	1091.2	1053.3	774.7				
	SPMS	1417.5	1226.3	1064.6				
	PPMS	1554.5	1265.1	1154.8				
	PMS	1480.2	1239.3	1134.1				
IL-10	HV+NIND	267.2	252.3	202.9	0.3021			
	OIND	170.2	215.4	72.8				
	RRMS	102.5	127.8	51.5				
	SPMS	191.6	249.3	95.1				
	PPMS	130.5	159.5	68.9				
	PMS	163.6	214.1	81.2				
VEGF-A	HV+NIND	2.2	3.3	1.1	0.7334			
	OIND	2.1	4.4	0.9				
	RRMS	10.1	39.2	0.9				
	SPMS	1.9	5.7	0.9				
	PPMS	2.2	7.7	0.9				
	PMS	2.1	6.7	0.9				
VEGF-C	HV+NIND	420.7	349.4	399.2	0.0293	OIND vs. RRMS	0.0422	−121.2
	OIND	377	345.0	346.1				
	RRMS	498.2	274.6	401.8				
	SPMS	394.2	214.1	373.3				
	PPMS	369.0	203.2	408.1				
	PMS	382.6	208.4	379.7				
	SPMS	0.6	1.7	0.1				
	PPMS	0.7	1.2	0.1				
	PMS	0.6312	1.5	0.1				
IL-6/IL-10	HV+NIND	2.3	3.5	0.6	0.9272			
	OIND	4.5	8.4	0.7				
	RRMS	3.9	6.9	1.7				
	SPMS	4.2	11.8	1.1				
	PPMS	12.4	28.2	0.9				
	PMS	7.9	21.25	0.9				
TNF-α/IL-10	HV+NIND	11.9	16.2	2.7	0.0916			
	OIND	40.5	103.5	7.4				
	RRMS	18.3	26.4	7.1				
	SPMS	170.0	1017.5	9.9				
	PPMS	54.6	83.2	13.4				
	PMS	117.2	750.4	11.8				
GM-CSF/IL-10	HV+NIND	1.0	1.5	0.4	0.2608			
	OIND	11.6	63.1	1.1				
	RRMS	8.8	30.2	1.1				
	SPMS	60.7	363.3	0.8				
	PPMS	7.1	12.4	3.2				
	PMS	36.2	267.9	1.7				
LT-α/IL-10	HV+NIND	8.1	9.2	5.0	0.0212	HV+NIND vs. PPMS	0.0135	−51.1
	OIND	32.5	86.5	10.7		HV+NIND vs. PMS	0.0292	−91.9
	RRMS	28.9	50.9	9.8				
	SPMS	134.5	784.7	9.1				
	PPMS	59.2	114.9	15.7				
	PMS	100.0	581.8	12.7				
VEGF-A/IL-10	HV+NIND	0.02	0.02	0.01	0.6044			
	OIND	0.2	0.9	0.01				
	RRMS	20.9	91.6	0.02				
	SPMS	2.2	15.4	0.01				
	PPMS	0.1	0.1	0.01				
	PMS	1.2	11.35	0.01				
VEGF-C/IL-10	HV+NIND	4.7	9.9	1.7	0.0085	OIND vs. RRMS	0.0210	−183.7
	OIND	10.1	25.7	2.7				
	RRMS	193.8	650.9	7.5				
	SPMS	38.4	190.9	3.3				
	PPMS	18.7	44.5	2.9				
	PMS	29.4	143.5	3.2				

First, we validated our hypothesis that undetectable levels of IL-10 and IL-6 in the pilot cohort was consequence of the selected ICCS procedure, as both cytokines were readily detectable by ELISA.

To assure that no bias was introduced by analyzing more than 1 CSF BCL per subject, we added a random effects term for each subject to account for variance that is due to repeated measures (i.e., multiple BCL) on a single subject. When we normalized cytokine production per 1 million of live, EBV-transformed CSF B cells we observed lower secretion of IL-10 in BCL derived from RRMS patients compared to non-inflammatory controls (difference between means [DM] = 164.7 pg/mL), but it did not reach statistical significance after adjusting for multiple comparisons (Figure 4A). Similarly, we saw no difference among diagnostic categories in the concentrations of IL-6 (Table 3).

From cytokines that were not measured in the pilot cohort, we found no significant differences among diagnostic categories in the B-cell secretion of GM-CSF (Table 3). Surprisingly, we observed lower IL-1β production in B cells derived from RRMS patients compared to all diagnostic categories (Figure 4B; OIND DM = 5.7 pg/mL, *p* = 0.0434; SPMS DM = 5.3, *p* = 0.0377; PPMS DM = 6.1 *p* = 0.0111; PMS DM = 5.6 pg/mL, *p* = 0.0028), as well as HV+NIND (DM = 14.4, *p* = 0.0031).

The validation cohort data revealed that increased VEGF secretion in MS B cells observed in the pilot cohort was limited to VEGF-C (Figure 4C). Furthermore, increased VEGF-C secretion was only observed in RRMS patients against all other diagnostic categories, even though only comparison with OIND reached statistical significance (DM = −121.2 pg/mL, *p* = 0.0422).

The validation cohort again mirrored the pilot cohort ICCS data with B cells derived from PMS patients secreting highest concentrations of TNF-α in comparison to RRMS, OIND, and non-inflammatory controls, although only comparisons to RRMS (Figure 4D; DM = −503.1 pg/mL, *p* = 0.0122) and non-inflammatory controls (Figure 4D; DM = −117.0 pg/mL, *p* = 0.0217) reached formal statistical significance. Similar data were obtained for LT-α, where the remaining significant difference was observed between PMS and RRMS (Figure 4E; DM = −389.0, *p* = 0.023).

Finally, because ratios of pro-inflammatory cytokines such as TNF-α and LT-α to immunoregulatory cytokine IL-10 were shown to be increased in peripheral blood of MS patients as compared to HV (34), we also generated analogous ratios for all pro-inflammatory cytokines we tested in the validation cohort. We observed a trend for lower ratios of pro-inflammatory cytokines to IL-10 in HV+NIND group, in comparison to subjects with intrathecal inflammation (i.e., OIND + RRMS + PMS). However, only the LT-α/IL-10 ratio achieved formal statistical significance with HV+NIND when compared to PPMS (Figure 4F; DM = −51.1, *p* = 0.0135) and PMS (Figure 4F; DM = −91.9, *p* = 0.0292). VEGF-C/IL-10 was higher in the RRMS cohort compared to OIND (Figure 4G; DM = −183.7, *p* = 0.0210).

### Exploring pathogenic role of CSF B cell-secreted cytokines

**Figure 5:**
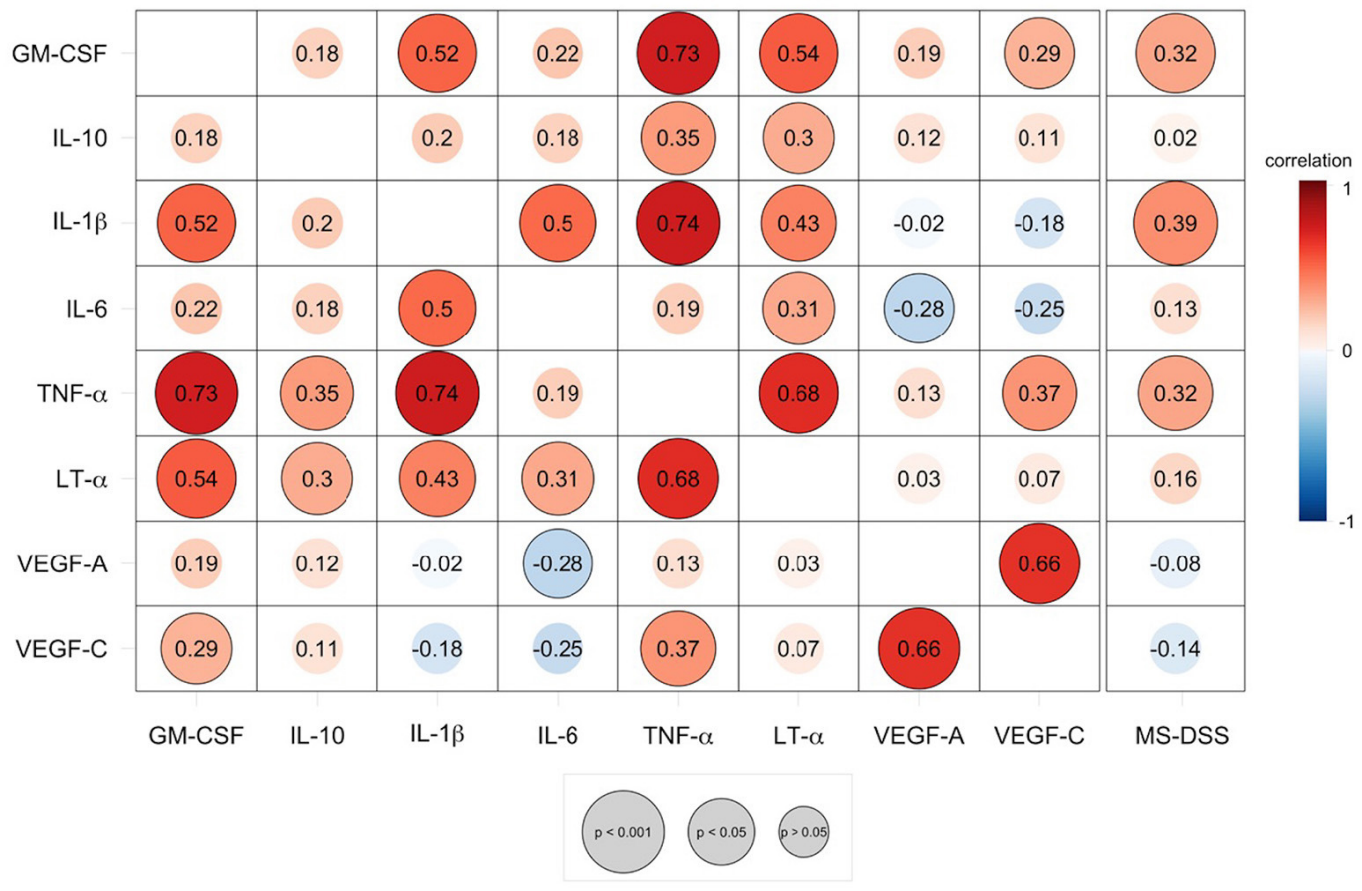
Speaman correlation matrix of validation group CSF BCL cytokine secretion and correlation of cytokines against MS disease severity scale (MS-DSS). Cytokines analyzed in the validation cohort are compared to each other and to the MS-DSS, which measures rates of disability progression. Red colors indicate positive, direct correlation, while blue colors indicate negative, inverse correlation, according to the continuous heatmap legend depicted at the right side of the correlation matrix. The Spearman correlation coefficient for each pair of cytokines is shown at the intersecting x- and y-axis label within each circle. Outlined circles represent significant correlations (*p* < 0.05 after adjustments for multiple comparisons) and increasing diameter of circles indicate a higher level of significance, according to the gray legend depicted below the correlation matrix.

As demonstrated by the correlation matrix (Figure 5), the validation cohort validated significant positive correlation between TNF-α and LT-α. TNF-α also strongly correlated with two additional cytokines with putative pathogenic role in MS, tested only in the validation cohort, GM-CSF and IL-1β In contrast, VEGF-C secretion was only positively correlated with VEGF-A secretion.

To explore whether any of the differences identified between MS subjects and controls participate in CNS tissue destruction, we assessed correlation between patient-specific CSF B cells cytokine production measured by ELISA (computed as average concentration of specific cytokine derived from all BCL isolated from specific subject) and new, sensitive model of MS severity, MS-DSS (29). MS-DSS is derived from statistical learning. In contrast to an older measure of MS severity, the Expanded Disability Status Scale (EDSS)-based MS Severity Score (35), MS-DSS can predict future rates of disability progression. In MS-DSS, the disability measurement by insensitive, discrete EDSS is replaced by a much more sensitive, statistical learning optimized continuous disability scale, Combinatorial Weight-Adjusted Disability Scale [CombiWISE (36)]. Additionally, disability ratings in MS-DSS are adjusted for patient-specific treatments using mathematical models derived from previously published meta-analysis of randomized clinical trials of disease-modifying treatments of MS (37). Finally, the MS-DSS model also contains certain demographic data and a Composite MRI Scale of CNS tissue destruction [COMRIS-CTD (38)]. MS-DSS is freely available via a web-based interphase at: https://bielekovalab.shinyapps.io/msdss/.

We observed mild, but highly significant correlations between MS-DSS and three correlated cytokines, IL-1β, TNF-α, and GM-CSF (Figure 5).

## Discussion

In this study, we present the first functional analysis (i.e., secretion of multiple cytokines) of intrathecal B cells in MS. Demonstration that related B cell clones populate all CNS compartments in MS, including CSF (39), validates the evaluation of CSF B cells as a representative population. The strength of our study resides in the application of highly standardized protocols in a blinded fashion to a sizable number of MS patients and controls prospectively collected at a single institution. Critically, we also validated most important new findings in prospectively acquired, independent validation cohort. We consider application of different methodology in the validation cohort (i.e., ELISA as opposed to ICCS) also a strength of this study, because it demonstrates that the data are independent of the methodological constraints of applied assays. Our initial hesitancy to use ELISA assay for answering question about differences in cellular phenotypes stem from the fact that in contrast to ICCS, which outputs values proportional to the concentration of cytokines in each cell, ELISA measures sum of the cytokines secreted by all living cells in the culture. Therefore, ELISA results are crucially dependent on the number of seeded cells and manual cell counts may introduce noise in the measurement. We successfully mitigated this problem by developing flow-cytometry based, objective normalization of imputed cell numbers among all studied subjects. This normalization was performed before unblinding the diagnostic categories.

The natural weakness of our study resides in the inability to measure cytokine production in neither CNS B cells nor CSF B cells and relying on an EBV transformation and *in vitro* expansion step. Nevertheless, CSF biomarkers have been established as viable proxies for inflammation originating from the CNS (40) and correlate with disease pathologies in MS (41, 42). As we discussed before, analogous clonotypes of T and B cells were identified in CNS tissue and CSF of individual patients. In regard to EBV transformation, due to limited numbers of B cells in the human CSF irrespective of diagnostic categories studied (with exception of intrathecal B cell lymphoma, which was not studied here), no current technologies can provide functional information from *ex vivo* isolated CSF B cells. We cannot rule out a possibility that EBV transformation might have affected phenotype of derived BCL (43, 44). However, blinding and written SOPs assured that any bias that EBV transformation and *in vitro* manipulation may have introduced affected all subjects equally. Therefore, this cannot explain the observed (and validated) differences among diagnostic categories. The obtained results to a large degree mitigate aforementioned concern, because our study reproduced a key finding from previously published *ex vivo* analyses of primary CSF B cells (7, 8), such as significantly higher levels of the co-stimulatory molecule CD80 (but not CD86) in CSF BCL derived from MS patients in comparison to controls (22) and trend for lower production of IL-10 observed in peripheral blood B cells derived from MS patients in comparison to HV (34).

There are important novel findings identified in the current study. First, we observed an almost five-fold higher efficiency of EBV transformation in MS patients compared to controls. While this may be partially driven by higher CSF pleiocytosis and higher proportion of CSF B cells in MS as compared to NIND patients and HV, this cannot be true for the OIND cohort, because OIND and MS subjects have similar proportions of CSF B cells (7, 8, 24). Furthermore, we observed only a weak correlation between CSF pleiocytosis or CSF IgG index and transformation efficiency, indicating that factors other than a higher number/proportion of CSF B cells in the MS cohort must underlie the differences in transformation efficiency. Higher transformation efficiency cannot be caused by higher EBV seropositivity (15), as we observed that all subjects on whom EBV serology data are available were seropositive (Table 1). Thus, higher transformation efficiency in MS could be mechanistically linked to higher intrathecal EBV expression (12) previously reported in MS, or may result from a defect of cytolytic activity of CSF T cells toward EBV transformed B cells, as described for peripheral T cells in MS (45). We consider the latter possibility less likely because use of cyclosporine A in our transformation protocol should limit the effect of cytotoxic T cells. The final possibility is that MS susceptibility genes enhance EBV infection of B cells and the rates of their immortalization. This hypothesis is consistent with both experimental data presented here and epidemiological data linking EBV to MS.

The second group of novel findings relate to MS-specific differences in cytokine phenotypes of intrathecal B cells. The first finding is rather surprising, the decrease in secretion of pro-inflammatory cytokine IL-1β in the CSF B cells from RRMS subjects in comparison to all other diagnostic subgroups. This finding was observed only in the validation cohort because we have not studied IL-1β secretion by ICCS in the pilot cohort. However, the level of statistical significance and congruency of findings against all control groups make this finding rather robust, even in the absence of independent validation. Increased IL-1β secretion is a hallmark of genetic auto-inflammatory syndromes associated with the activation of the inflammasome, predominantly in the cells of innate immunity. IL-1β secretion is regulated by caspases, especially by caspase-1, but IL-1β secretion is unconventional in that it does not enter the classical endoplasmic reticulum (ER)-Golgi protein secretion pathway. Instead, its secretion requires formation of a new intracellular compartment, called Unconventional Protein Secretion (UPS) (46), induced by stress such as the deprivation of nutrients (47). Additionally, IL-1β secretion is linked to autophagy, raising a possibility that the unique cytokine secretion phenotype of RRMS B cells may be linked to dysfunction in autophagy pathways, previously identified in immune cells of MS and its animal model Experimental Autoimmune Encephalomyelitis (EAE) (48). However, we must not forget that ELISA measures the difference between secretion and consumption and that IL-1β is also consumed by B cells, especially of the regulatory phenotype (49). Therefore, enhanced consumption of IL-1β by B cells derived from RRMS patients, due to their higher proportion of regulatory B cells in the intrathecal compartment represents an emerging new hypothesis, testable in future studies. Intriguingly, IL-1β production was highly correlated with the secretion of TNF-α and LT-α and these cytokines were not only expressed at higher levels in PMS but also correlated with MS severity measured by MS-DSS. In other words, patients with higher B cell secretion of IL-1β, TNF-α (and GM-CSF) have more aggressive MS, characterized by faster accumulation of disability. Due to collinearity between these three cytokines, it is unclear whether each has an independent contribution to MS severity. We tried to answer this issue by exploring statistical learning using either generalized boosting machine or multiple linear regressions to assess whether a complex model that contains all three (or two) of these cytokines correlates better with MS-DSS than individual cytokines. None of these models performed better than IL-1β alone (data not shown), suggesting that IL-1β secretion has the dominant effect on MS severity. While correlation is not causation, our results generate the hypothesis for future studies that B cell secretion of IL-1β and/or their decreased consumption of IL-1β due to low proportion of regulatory B cells in the intrathecal compartment contributes to CNS tissue destruction in MS.

The remaining differences in the phenotype of MS intrathecal B cells were observed in both pilot and validation cohorts. We found and validated the enhanced production of VEGF, specifically the VEGF-C (but not VEGF-A) family member, in RRMS patients. VEGF-A binds to VEGF receptors 1 (VEGFR1; Flt-1) and−2 (VEGFR2; KDR/Flk-1), expressed predominantly in endothelial cells (50). Not surprisingly, then, VEGF-A mediates the effects of VEGF on endothelial cells, stimulating angiogenesis, but also opening epithelial tight junctions, leading to vascular edema. In contrast, VEGF-C binds a third receptor (VEGFR3; Flt4), expressed on the cells of the lymphatic endothelium, and thus mediates lymphangiogenesis (50). At this point, we can only speculate about functional consequences of enhanced VEGF-C secretion by RRMS B cells; it is possible that the lymphangiogenesis initiates the formation of lymphoid aggregates, also called tertiary lymphoid follicles, found in the meninges of patients with long-standing MS and being linked to more severe CNS tissue destruction (11).

We also found (and validated) that, compared to RRMS patients' BCL, intrathecal B cells derived from PMS patients produced higher amounts of two closely related cytokines, LT-α and TNF-α. In view of the VEGF-C findings described above, it is important to note that both LT-α and TNF-α play an important role in the development and maintenance of tertiary lymphoid follicles (10, 11). Thus, the major conclusion we can derive from our analysis of the functional phenotype of CSF B cells is that B cells derived from MS patients differ from B cells derived from non-MS controls, including those with inflammatory neurological diseases in their pro-lymphangiogenic potential. Why VEGF-C is upregulated only in B cells derived from the early, RRMS stage, and why B cells from PMS instead secrete higher concentrations of TNF-α and LT-α are intriguing topics for future investigation. Similar MS-stage-specific differences were observed in peripheral blood for a subset of MS susceptibility genes, using expression profiling (52). Specifically, previously reported MS susceptibility gene *MYC* (51) was upregulated in peripheral blood only in RRMS, but not PMS patients (52). Even further, mice overexpressing MYC in B-cell progenitors had increased VEGF production and concurrent stimulation of angiogenesis and lymphangiogenesis (53). It is possible that *MYC* deregulation plays a causal role in the observed VEGF-C phenotype in RRMS, but not progressive patients. In contrast, PMS groups (but not RRMS patients) had significantly lower expression of TNFRSF1A (52) in blood leukocytes, suggesting that this is either a consequence of higher TNF-α production in progressive MS (as part of negative feedback loop), or, alternatively, lower expression of the receptor may cause lower consumption of TNF-α by immune cells derived from progressive MS. Clearly, expression or genes and translation of proteins can change with disease evolution, underlying processes such as terminal differentiation of memory B cells induced by chronic inflammation, as has been previously described for T cells in PMS but not RRMS (54). Alternatively, the observed changes may have a causative role: e.g., patients with increased production of TNF-α and LT-α may be predisposed to progressive disease. These two possibilities can be only addressed in longitudinal studies that will assess the rate of MS progression in RRMS subjects whose intrathecal B cells produce high or low levels of TNF-α and LT-α.

In conclusion, the observed results suggest that the defining role intrathecal B cells play in MS (in contrast to other CNS inflammatory diseases) is to facilitate compartmentalization of the inflammation into CNS tissue by promoting lymphangiogenesis. The role of EBV infection in this process remains unknown.

## Data availability statement

All data generated or analyzed during this study are included in this article and its Supplementary Information files.

## Author contributions

BB conceived and supervised the study. QX conducted the ICCS and JS conducted the ELISA experiments. QX, ER, and SW optimized the conditions for the B cell immortalization and immortalized the cells, cryopreserved, and thawed CSF B cells. JS and ER optimized the PMA/IoM stimulation of B cells for the validation cohort. PK developed and maintained the database. KJ, QX, JS, TW, and BB analyzed the results. QX, JS, SW, KJ, and BB drafted the paper. All authors reviewed the manuscript.

### Conflict of interest statement

The authors declare that the research was conducted in the absence of any commercial or financial relationships that could be construed as a potential conflict of interest.
